# Repurposing Didanosine as a Potential Treatment for COVID-19 Using Single-Cell RNA Sequencing Data

**DOI:** 10.1128/mSystems.00297-20

**Published:** 2020-04-14

**Authors:** Fadhl M. Alakwaa

**Affiliations:** a Department of Neurology, University of Michigan, Ann Arbor, Michigan, USA; University of California San Diego

**Keywords:** COVID-19, drug, repurposing

## Abstract

As of today (7 April 2020), more than 81,000 people around the world have died from the coronavirus disease 19 (COVID-19) pandemic. There is no approved drug or vaccine for COVID-19, although more than 10 clinical trials have been launched to test potential drugs. In an urgent response to this pandemic, I developed a bioinformatics pipeline to identify compounds and drug candidates to potentially treat COVID-19. This pipeline is based on publicly available single-cell RNA sequencing (scRNA-seq) data and the drug perturbation database “Library of Integrated Network-Based Cellular Signatures” (LINCS). I developed a ranking score system that prioritizes these drugs or small molecules. The four drugs with the highest total score are didanosine, benzyl-quinazolin-4-yl-amine, camptothecin, and RO-90-7501. In conclusion, I have demonstrated the utility of bioinformatics for identifying drugs than can be repurposed for potentially treating COVID-19 patients.

## Abstract (precis)

As of today (7 April 2020), more than 81,000 people around the world have died from the coronavirus disease 19 (COVID-19) pandemic. There is no approved drug or vaccine for COVID-19, although more than 10 clinical trials have been launched to test potential drugs. In an urgent response to this pandemic, I developed a bioinformatics pipeline to identify compounds and drug candidates to potentially treat COVID-19. This pipeline is based on publicly available single-cell RNA sequencing (scRNA-seq) data and the drug perturbation database “Library of Integrated Network-Based Cellular Signatures” (LINCS).

## OPINION/HYPOTHESIS

As of today (7 April 2020), more than 81,000 people around the world have died from the coronavirus disease 19 (COVID-19) pandemic. There is no approved drug or vaccine for COVID-19, although more than 10 clinical trials have been launched to test potential drugs. These potential drugs have included drugs based on their promising effects against severe acute respiratory syndrome (SARS) or Middle East respiratory syndrome (MERS) or on their ability to block host target proteins, such as angiotensin-converting enzyme 2 (ACE2), which may be a SARS receptor (1). Bioinformaticians and data scientists can help by identifying potential candidates from available big data to narrow the scope of research and accelerate discovery. Focusing efforts on repurposing drugs already approved for other applications and with known safety profiles saves on cost, shortens the time to approval, and accelerates the bench-to-bedside time, bringing aid to COVID-19 patients sooner.

In an urgent response to this pandemic, I developed a bioinformatics pipeline to identify compounds and drug candidates to potentially treat COVID-19. The results from this pipeline are still preliminary and have not been validated *in vitro*. This pipeline is based on publicly available single-cell RNA sequencing (scRNA-seq) data (2) and the drug perturbation database “Library of Integrated Network-Based Cellular Signatures” (LINCS) (3). The scRNA-seq data contains samples from eight healthy human lung transplant donors and eight samples from patients with pulmonary fibrosis (Gene Expression Omnibus repository accession no. GSE122960) (2). Analyzing this scRNA-seq data reveals that ACE2 is mainly expressed in type II alveolar cells (AT2) cells. I first identified significantly differentiated genes (DEGs) (adjusted *P* value of <0.05) between AT2 cells that express ACE2 and AT2 cells that do not express ACE2. I found 30 upregulated DEGs, including SLC1A5, CXADR, CAV2, NUP98, CTBP2, GSN, HSPA1B, STOM, and RAB1B. Downregulating these genes will be very important in a treatment approach because they regulate viral reproduction and transmission (4).

Next, I used the Connectivity Map Linked User Environment (CLUE) platform, which connects to the LINCS database of small-molecule perturbations on gene expression, to identify drugs and compounds that can reverse these upregulated genes (3). CLUE selected 39 out of 2,837 drugs with a negative connectivity score (CS) of less than −90. The closer the CS is to −100, the greater the chance the drug has of reversing upregulated DEGs, in this instance, drugs that can reverse expression of DEGs upregulated in ACE2-expressing AT2 cells. The complete drug list can be downloaded from https://github.com/FADHLyemen/COVID-19. None of these drugs have been validated. Third, I developed a ranking score system that prioritizes these drugs or small molecules based on three developed scores, the CS from CLUE (S1), the genetic perturbation score (S2), and the class score (S3). The definitions and equations of these scores can be found at https://github.com/FADHLyemen/COVID-19. The four drugs with the highest total score (St) are didanosine, benzyl-quinazolin-4-yl-amine, camptothecin, and RO-90-7501 (Table 1).

**TABLE 1:** A list of potential drugs for treating COVID-19 based on scRNA-seq and LINCS database

Drug class	Drug name	Target(s)	Connectivity score (S1)	Genetic perturbation score (S2)	Class score (S3)	Total score (St)
Nucleoside reverse transcriptase inhibitor	Didanosine	PNP	−91.84	−99.92	−100.0	−291.76
EGFR inhibitor	Benzyl-quinazolin-4-yl-amine	EGFR	−98.87	−98.65	−73.91	−271.43
Topoisomerase inhibitor	Camptothecin	TOP1, HIF1A	−96.86	−77.40	−93.75	−268.01
Beta amyloid aggregation inhibitor	RO-90-7501	APP	−99.61	−92.89	−75.00	−267.50

Didanosine is an HIV antiviral drug that belongs to the nucleoside reverse transcriptase inhibitor class (5). All the drugs belonging to this class have negative scores (S3). In addition, the CS of knocking down purine nucleoside phosphorylase (PNP), the target gene of didanosine, is also negative (S2). Didanosine is a prescription drug approved by the U.S. Food and Drug Administration (FDA) for treating HIV infection. The second, benzyl-quinazolin-4-yl-amine, is a compound that belongs to the family of epidermal growth factor receptor (EGFR) inhibitors. Interestingly, knocking down EGFR also produced a negative score. The third molecule on our list is camptothecin, a topoisomerase inhibitor and alkaloid present in Camptotheca acuminata, which is used in traditional Chinese medicine. Knocking down camptothecin’s target gene also generates negative scores, such as to TOP1 (−52) and TIF1A (−92). Sixteen out of 17 (93%) drugs belonging to the class of topoisomerase inhibitors have negative scores (S3). The last drug, RO-90-7501, targets the amyloid precursor protein (APP) gene and is an amyloid-β42 aggregation inhibitor and candidate Alzheimer’s disease molecule (6). In conclusion, I have demonstrated the utility of bioinformatics for identifying drugs that can be repurposed for potentially treating COVID-19 patients. The drugs listed are preliminary and need *in vitro* validation. I also call for such approaches on COVD-19 targets other than ACE2 to nominate additional candidates for *in vitro* and *in vivo* testing, which could accelerate drug discovery for COVID-19.
